# Managing acute pain in HIV+/AIDS patients: knowledge and practice trends among emergency physicians of major tertiary care centers of a developing country

**DOI:** 10.1186/s13104-020-05095-1

**Published:** 2020-05-26

**Authors:** Aliya Ahmed, Gauhar Afshan, Robyna Irshad Khan, Badar Afzal, Seemin Jamali, Nighat Farooq, Sarosh Saleem, Rubaba Naeem, Uzma Khan

**Affiliations:** 1 Department of Anaesthesiology, 2nd-Floor Private Wing, Aga Khan University, Stadium Road, P.O. Box 3500, Karachi, 74800 Pakistan; 2 Department of Emergency Medicine, Aga Khan University, Karachi, Pakistan; 3 Accident and Emergency Department, Jinnah Postgraduate Medical Centre, Karachi, Pakistan; 4 Accident and Emergency Department, Civil Hospital Karachi, Karachi, Pakistan; 5 Paediatrics Department, Indus Hospital, Karachi, Pakistan

**Keywords:** Pain management, HIV, AIDS, Emergency physicians, Acute pain

## Abstract

**Objective:**

To assess knowledge and practice trends in managing acute pain in patients infected with human immunodeficiency virus (HIV+) or having acquired immunodeficiency syndrome (AIDS) among emergency physicians of four tertiary care hospitals. Acute pain management in such patients is complex because of multiple concomitant painful conditions related to their disease. After obtaining ethical approval and written informed consent, emergency physicians were requested to fill out a questionnaire.

**Results:**

Out of 84 physicians who participated, 49 had managed HIV+/AIDS patients during the preceding year. Out of the 49, 30 (61.2%) physicians stated that they used a combination of analgesics for acute pain in these patients. Forty-two (50%) out of the 84 participants believed that routine doses of opioids were adequate for pain relief, while 42 (50%) agreed that pain management was more complex in these patients mainly due to presence of multiple coexisting problems and psychological issues. Only 26 (31%) respondents considered that pain was under-reported and under-treated in these patients, mainly because physicians were more focused on patients’ other disease related complications and issues. Formulation of guidelines are recommended for effective acute pain management in these patients encompassing associated issues, including concomitant painful conditions, opioid dependence, psychiatric problems, etc.

## Introduction

Despite considerable research on pain management in recent decades, inadequate acute pain control is still a sizable problem, even more so in low- and middle-income countries [1]. Some patient groups tend to be affected more in this respect, and patients infected with human immunodeficiency virus (HIV+) or suffering with acquired immune deficiency syndrome (AIDS) are among such patients [2].

Acute pain management in HIV+/AIDS patients is a complex task because of multiple concomitant painful conditions, for instance cellulitis, septic arthritis, abscesses, HIV+/AIDS related neuropathies, etc. [2, 3]. A number of these patients may be addicted to opioids [4], which further complicates acute pain management. Moreover, HIV+/AIDS patients are reported to be at a higher risk of being involved in accidents and trauma [3, 5, 6], leading to these patients presenting at the emergency wards with moderate to severe acute pain. All these factors mandate that physicians responsible for managing these patients in emergency situations are knowledgeable about the complex nature of their pain, and are able to address it effectively and safely. In order to standardize acute pain management in these patients, formulation of guidelines is fundamental, keeping in mind the available resources. Guidelines are developed with the intent of outlining the best practices. Currently there are no acute pain guidelines or recommended best practices that cover the wide range of pain experiences across all patient populations, especially vulnerable populations such as patients living with HIV+/AIDS who experience ongoing pain due to multiple reasons despite the advances in treatment of the disease itself. Separate guidelines for acute pain management are highly important for these patients because, when managing their acute pain, consideration needs to be given to other ongoing issues such as opioid-dependence, psychosocial problems, etc., due to which routine analgesics in usual doses would not relieve their pain effectively.

According to an estimate by The Joint United Nations Programme on HIV/AIDS (UNAIDS), there are approximately 130,000 people living with HIV in Pakistan [7]. Several factors make Pakistan vulnerable to HIV spread, low literacy, high poverty, paucity of basic health facilities and unsafe blood transfusions being the most prominent factors [7, 8]. Towards the goal of developing acute pain management guidelines for HIV+/AIDS patients, assessment of baseline knowledge of physicians responsible for their management and their current practice trends is an integral first step. This survey was conducted to assess the knowledge and practice trends in managing acute pain in HIV+/AIDS patients among emergency physicians of four tertiary care hospitals of a developing country. The reason for including emergency physicians was that they are responsible for the first-line management of patients presenting with acute pain due to trauma or other acute illnesses.

## Main text

### Methods

A cross-sectional survey was conducted at four tertiary care hospitals of a major city of a developing country after approval from Ethics Review Committees and individual written informed consent from participants. Two of the hospitals were 300 bedded and 500-bedded private hospitals, while two were large government hospitals with 1500-beds each. Emergency physicians, consultants and trainees with an experience of more than 1 year were included.

A questionnaire was developed by the authors comprising of anaesthesiologists and emergency physicians. The questions pertained to physicians’ knowledge and practice about managing moderate to severe acute pain in HIV+/AIDS patients. In view of the research findings that HIV still carries a stigma in the authors’ country [9] and that pain management in emergency rooms and general physicians’ clinics is often suboptimal [10, 11], two open-ended questions were included in the questionnaire. The aim was to explore the perception of the participants about the probable reasons for the under treatment and under reporting of pain and complex nature of pain in HIV+/AIDS patients and thus to assess their viewpoint about the sociocultural aspects that are influencing pain management in this group of patients.

The questionnaire was shared with two senior consultants of anaesthesiology and emergency medicine for feedback and suggestions. Following this five pilot sessions were conducted with senior emergency medicine trainees. Based on the feedback and finding of these sessions, survey questions were reviewed and modified. The questionnaire was administered by a research assistant trained by the primary investigator, who visited the study sites during each duty shift, obtained informed consent, distributed the questionnaires and collected filled out questionnaires after 1–2 h. The identity of participants was kept confidential by assigning a code number to each participant and each hospital. The filled out questionnaires were kept under lock and key and data were entered in a password-protected computer. Data were entered and analyzed using statistical packages for social science version 19 (SPSS Inc., Chicago, IL). All categorical variables were summarized as frequencies and percentages.

### Results

Eighty-four physicians participated in the survey, 25 (29.8%) consultants and 59 (70.2%) trainees with more than 1-year experience. Questions were divided into two categories: practice-related and knowledge-related. Only two physicians had managed more than 80 HIV+/AIDS patients during the preceding year, while 35 had not managed any such patient. Thirty (61.2%) physicians, out of the 49 who had treated HIV+/AIDS patients in the preceding year, stated that they use a combination of analgesics for relieving acute pain in HIV+/AIDS patients, while the rest use single analgesic therapy. More responses to practice-related questions are provided in Table 1.

**Table 1:** Responses to practice-related questions regarding the management of acute pain in HIV+/AIDS patients (responses by physicians who had managed HIV+/AIDS patients in the preceding year: n = 49)

Practice trends	Frequency	Percentage
Approximately how many HIV-infected patients have you treated in your emergency room during the last year?		
≤ 20	43	87.8
21 to 40	2	4.1
41 to 60	1	2.0
61 to 80	1	2.0
> 80	2	4.1
What was the most common presenting complaint?		
Trauma	6	12.5
Non-traumatic conditions	26	54.2
Pain due to other diseases	8	16.7
Others	8	16.7
No response	1	2.0
What drugs do you commonly use to treat acute pain in HIV+/AIDS patients?		
Opioid	9	18.4
NSAID	4	8.2
Paracetamol	4	8.2
Combination	30	61.2
Other	0	0
No response	2	4.1
What other modalities do you commonly employ to treat acute pain in HIV+/AIDS patients?		
Physiotherapy	13	26.5
Acupuncture	0	0
Psychotherapy	3	6.1
Nerve blocks	12	24.5
Regional anesthesia	9	18.4
Others	5	10.2
No response	7	14.3
Is pain assessment done for all patients presenting to the emergency room at your hospital?		
Yes	37	75.5
No	12	24.5
What method is used for assessment of pain in your emergency room?		
Categorical Scale (mild, moderate, severe)	18	36.
Visual Analog Scale	5	10.2
Numeric rating Scale	26	53.1
No response	0	0
Are any guidelines in place at your hospital for managing trauma related pain?		
Yes	28	57.1
No	20	40.8
No response	1	2.0
Is multi-modal therapy for management of acute pain part of HIV patients’ treatment in your unit?		
Yes	13	26.5
No	5	10.2
No response	31	63.3
Is pain treated with available resources to patient’s satisfaction?		
Yes	21	42.9
No	2	4.1
No response	26	53.1

Regarding knowledge-related questions, 42 (50%) out of the 84 participants claimed that the usual routine doses of opioids are adequate for effective pain relief in HIV+/AIDS patients presenting with acute pain. Responses to knowledge-related questions are provided in Table 2. Forty-two (50%) participants agreed that pain management is more complex in HIV+/AIDS patients. The most frequent reason provided was presence of multiple coexisting problems and psychological issues (Table 3). Only 26 (31%) respondents believe that pain is under-reported and under-treated in these patients. The main reason suggested for this was that both physicians and patients were more focused on other ongoing issues being faced by these patients (Table 3).

**Table 2:** Knowledge regarding management of acute pain in HIV+/AIDS patients (n = 84)

Questions related to knowledge	Frequency	Percentage
Are you familiar with World Health Organization (WHO) analgesic ladder?		
Yes	60	71.4
No	24	28.6
What is the dose of opioid drugs required to treat the pain related to traumatic injury in HIV-infected patients?		
Usual dose	42	50.0
More than the usual dose	23	27.4
Less than the usual dose	9	10.7
No response	10	11.9
Is management of pain more complex in HIV-infected patients?		
Yes	42	50.0
No	35	41.7
No response	7	8.3
Do you think pain is under-reported and under treated in HIV-infected people?		
Yes	26	31.0
No	51	60.7
No response	7	8.3

**Table 3:** Reasons provided for ‘Yes’ response to the open-ended questions

‘Do you think management of pain is more complex in HIV-infected patients?’ (n = 42)		
1. Chronic pain due to other HIV/AIDS related problems	9	21.4%
2. Drug dependence/addiction leading to decreased response to routine doses	5	11.9%
3. Associated psychological issues	5	11.9%
4. Multisystem involvement/multiple problems	13	30.9%
5. Immune deficiency	3	7.1%
6. Decreased pain threshold/need for higher doses	4	9.5%
7. No clear guidance available to healthcare professionals/decreased awareness	3	7.1%
‘Do you think pain is under-reported and under-treated in HIV infected people?’		
1. It is considered a stigma in society, due to which HIV/AIDS is itself under-reported, and patients may avoid sharing the agony of their pain	3	11.5
2. Patients may be on regular pain killers at home due to longstanding ongoing pain conditions and do not inform about new onset of acute pain	5	19.2%
3. Failure to enquire specifically about pain by physicians on each visit and duly address it	7	26.9%
4. Patients often have multiple issues going on and come to hospital with HIV/AIDS related complications rather than for isolated pain management, therefore do not specifically report pain	8	30.8%
5. Generally pain is under treated in all patients with moderate to severe pain as physicians do not feel comfortable in using opioids; multiple analgesics are given to avoid using high doses of opioids	3	11.5%

### Discussion

Pain relief is a basic human right. Pain in HIV+/AIDS patients has been recognized as a major global healthcare problem [12]. When these patients present with acute pain in emergency situations, they are often managed by physicians who do not encounter such patients routinely. This may lead to sub-optimal pain management and undue suffering.

Only two emergency physicians in our study had managed more than 80 HIV+/AIDS patients in the last 1-year, while majority of the respondents had managed less than 20 such patients (Table 1). This finding endorses our inference that emergency physicians do not manage such patients routinely. Patients having HIV+/AIDS are usually cared for by infectious disease physicians and for day-to-day issues they visit their primary-care physicians [12, 13]. Other physicians therefore may not be aware of the special issues related to their various co-existing painful conditions [2, 3], which make their acute pain management more challenging. This highlights the need for evidence-based guidelines in line with the locally available resources for effective management of acute pain in HIV+/AIDS patients [5, 14].

Around 61% of the respondents stated that they use a combination of analgesics for relieving moderate to severe acute pain in HIV+/AIDS patients. This is an encouraging response as multimodal analgesia is the recommended method for managing moderate to severe acute pain [15], using more than one analgesic drug to obtain additive beneficial effects with reduced side effects [13]. Almost 19% of the study participants stated that they use opioids alone for acute pain relief and a similar number claimed using a single non-opioid analgesic agent for this purpose (Table 1). As pointed above, HIV+/AIDS patients might be on long-term analgesic medications due to concomitant painful conditions [2, 3]. A single analgesic agent would therefore not be able to effectively relieve moderate to severe acute pain in these patients. Moreover, use of larger doses of a single analgesic agent is bound to cause troublesome side effects, especially when opioids are being used. Knowledge about multimodal analgesia and guidelines for effective use of various combinations of available analgesic agents and modalities are thus a pressing need.

Fifty percent of the participants claimed that the routine doses of opioids are adequate for HIV+/AIDS patients presenting with moderate to severe acute pain. This is a misconception because chronic pain is commonly present in these patients [3], for which they may already be using opioid agents. Moreover, a sizable number of these patients may be addicted to opioids [16]. Therefore, acute traumatic pain in such patients would not be relieved with routine doses of opioids and there would also be a risk of developing withdrawal symptoms. Associated psychiatric problems may further aggravate pain perception [5, 17]. Adjuvant therapies are therefore recommended in this group of patients to augment analgesia [12]. Although half of the participants did realize that acute pain management is not straightforward in HIV+/AIDS patients, they had no guidance available due to a lack of availability of practice guidelines for managing acute pain in these patients [18]. This lack of guidance makes treatment decisions difficult and leads to unnecessary patient suffering.

Thirteen participants stated that the reason for complexity in pain management in HIV+/AIDS patients was the multiple health problems that co-existed in these patients. This is a correct perception as these patients often suffer with peripheral neuropathy, painful dermatitis, infections, and other pain syndromes, thus having pain of varying severity in their daily lives [2, 12]. Five survey respondents identified psychological problems as a reason for difficulty in managing acute pain in HIV+/AIDS patients. Psychiatric comorbidities are known to co-exist in these patients, often attributed to various social obstacles faced by them [4, 5], making pain assessment and management more complex. Moreover, the high prevalence of trauma in HIV+/AIDS individuals is known to lead to post-traumatic stress disorder and dissociative symptoms [13]. Inadequate pain relief may further worsen patient’s disease-related depression and could have serious consequences, including ideation of suicide [17].

Only 31% respondents agreed that pain is under-reported and under-treated in HIV+/AIDS patients. Parker et al. have reported marked under-treatment of pain in HIV+/AIDS patients [2]. They have identified reasons for this under-treatment, including lack of awareness of pain as a separate entity to be addressed [19], lack of availability of strong analgesics [20], fear of addiction [19, 21], and lack of time for consultations [21]. Reasons identified for under-reporting of pain include lack of knowledge that treatments other than antiretroviral therapy are available, fear that pain may be a sign of serious complications, fear that the physician might get distracted if they inform about their pain, and fear of being considered a difficult patient [2]. Furthermore, the stigma still attached to HIV and AIDS is an important reason for under-reporting of pain [9].

Effective acute pain management depends upon multiple factors including the available resources. When using multi-modal analgesia involving strong analgesics, physicians in emergency department need to be aware of the side effects and complications involved and be prepared to diagnose and treat them. Optimal pain relief in these patients may require a multidisciplinary approach [5, 22]. In spite of considerable advances in the management of HIV/AIDS in recent years, there are no guidelines available for management of acute pain in these patients. Formulation of practical guidelines for acute pain management in these patients would be very useful in optimizing their pain relief.

### Conclusion

We discovered considerable gaps in emergency physicians’ knowledge regarding acute pain management in HIV+/AIDS patients. Best approach is to use a multimodal analgesic regime modified according to the available resources. The authors strongly recommend formulation of guidelines for effective acute pain management in these patients, encompassing associated issues, such as concomitant chronic pain conditions, opioid dependence, psychiatric problems, etc.

## Limitations

A limitation of our survey is that, in addition to consultants, we have included trainee emergency physicians with more than 1-year experience. The reason for this was that trainees are usually the first respondents when patients present in the emergency room following acute trauma and other acute conditions and therefore manage the patients at the time of maximum pain intensity. They should therefore be knowledgeable about the specific concerns regarding acute pain management in this patient population.

## Data Availability

All data generated and analysed during this study are included in this published article and the additional tables. The datasets used and analysed during the current study are available from the corresponding author on reasonable request.

## Abbreviations

HIV: Human immunodeficiency virus
AIDS: Acquired immunodeficiency syndrome
